# Finite element analysis of RC beams using static experimental data to predict static and dynamic behaviors

**DOI:** 10.1038/s41598-024-82537-x

**Published:** 2024-12-28

**Authors:** Arvindan Sivasuriyan, D. S. Vijayan, Naveen Sankaran, D. Parthiban

**Affiliations:** 1 Institute of Civil Engineering, Warsaw University of Life Sciences—SGGW, 02-787 Warsaw, Poland; 2 Civil Engineering, Aarupadai Veedu Institute of Technology (AVIT), Vinayaka Missions Research Foundation (VMRF), Paiyanoor, Chennai, 603104 India; 3 DACK Consulting Solutions, Inc, White Plains, NY 10601 USA

**Keywords:** FEA, MEMS, FRS, PZS, RC Beam, Dynamic, Analysis, Civil engineering, Mechanical engineering

## Abstract

This study comprehensively compares dynamic and static forces in reinforced concrete (RC) beams, utilising experimental and finite element analysis (FEA) methodologies. Experimental tests involve monotonic two-point loading of 1 m x 150 mm x 150 mm RC beams using a universal testing machine (UTM). Deflection measurements are taken at three distinct locations (S1–S3) using various sensors, including force resisting sensor (FRS), flex sensor (FLS), MEMS accelerometer, and Piezoelectric sensors. The experimental data is then compared with FEA results obtained through ANSYS 16.0 software. Additionally, dynamic analysis is conducted, and results are presented in 3D graphical format. Mode shapes and harmonic responses are analysed. The study further discusses the sensor outcomes that align closely with FEA results. Overall, this research provides valuable insights into the dynamic and static behaviour of RC beams and offers a robust validation approach through experimental and computational analyses.

## Introduction

Rapid urbanisation across the globe has made concrete the most suitable material for construction work around the world. Based on the upgradation and convolution in the construction domain have made it even more challenging. Reinforced concrete (RC) admits every requirement to handle geometry into practical realisation. This cement (Brittle nature) structure, once bonded with metal rods (Ductile nature), provides better compressive strength and tensile strength, respectively. These RC structures undergo Static and Dynamic loading conditions in all possible ways. A splendid amount of research has been carried out in analysing static and dynamic loading in different traditional ways related to Eigenvalues and numerical and analytical methods. Some of these methods during analysis may or may not converge the data as per requirements. In such a precarious state of affairs, it is necessary to introduce sensors with required variables into RC structures to manage and understand them effectively, thus providing better accessibility in maintenance and prolonging structures’ lifetime. S.W.Doebling et al.^1^ have made early remarks on various nondestructive ways to examine civil structures similar to metal structures, using ultrasound, x-rays, Acoustics and radiography to detect the damage caused in structures in the overall area. At the same timeline, there were research works carried out on Wavelet theories for a better understanding of crack forming and propagation by K.Liew et al.^2^, where identification of cracks on supported beam was made using traditional methods like Eigen theory and wavelet methods to converge the analysis. Outcomes precisely show that the Eigenvalue doesn’t lead to the required results but closes in results by the wavelet method.

In the late 20th century, they evolved with scarce literature on the Laser Doppler Vibrometer as a new emerging technology in understanding and analysing the patterns of structural damage observations. P.Sriram et al.^3^ made fascinating efforts in simulating Multiple discrete ways of sensing various distributed loads across the structures taken up for the test. In the same timeframe, work was even carried out on optimisation techniques of data accession from various objectives of dynamic loading. C.E. Seeley et al.^4^ and B.T.Wang et al.^5^ have made compelling remarks on multi-objective optimization on design objectives of piezoelectric sensor usage for understanding natural frequency and resonance by optimising and capturing mechanical vibration caused by civil structures in the form of Dissipating energy and Piezoelectric transducer in cantilever beam structure for modal testing respectively.

Localised structural damage detection is considered more prime than global damage detection, and the early 21st century paved the way for various possibilities in identifying local damage and improvising it in its core level detection of crack development and propagation. C. C. Chang et al.^6^ considered works on detecting local damage on Timoshenko Beam using Spatial Wavelet Analysis. The crack position was accurately identified based on understanding the distribution of Wavelet Coefficient and Decomposition of mode shape across the Timoshenko beam. Some literature work by Shi et al.^7^ suggests even incomplete mode shapes also influence finding local structural damage in RC beams by using the Sensitive Statistical Method and upgrading it with measured Natural Frequency. S. Bhalla et al.^8^ observed dynamic loading based on various aspects using electric impedance, capturing effective resonant frequency. It was remarked that ingress of electricity directly proportionates to increases in temperature. Spring mass damper (SMD) precisely captures and calibrates impedance across the structure. The conservation of civil structures demands nondestructive maintenance, which revolutionised the understanding of inherent parameters influencing ununiform loading. Orak et al.^9^ carried out various works in non-destructive testing (NDT) and implied it in construction.

His works on concrete structures’ dynamic characterisation use various nomenclatures like dynamic modulus of rigidity, dynamic modulus of elasticity, and dynamic poisson’s ratio. Studies on improving the tensile, durability and strength of RC structures are very much required by age. XU. B.W et al.^10^ did various works in identifying the best suitable fibres to achieve enhanced properties of the tensile nature of the RC structure, which led to a whole new study combination between fibre reinforcement and its observation of properties through external analysis.

These works were later carried out effectively using the finite element method by Dahmani et al.^11^ for spotting cracks in RC beams using ANSYS Software; the sequel of crack formation and its response in occurrence and propagation are established at the elementary level. The author developed a three-dimensional non-linear finite element analysis of RC beams using SOLID65 solid element upon analysing the nonlinear material model and splotch reinforced model to identify concrete cracks and the mechanical behaviour of implanted steel. Similar analytical and experimental outcomes were also observed and hand-calculated. Insight criteria were critical crack regions, load and deflection at various loading conditions in RC beams. Based on the comparison, both the results were more or less similar to the results.

Y.Xla et al.^12^ suggested that even temperature changes affect material properties, which involves the caste of the piezoelectric sensors to read signals of susceptible temperature variation, which is amicable towards structural damages. Concrete heat distribution is generally non-uniform and time-dependent even when subjected to a similar temperature environment. Banu D et al.^13^ tested static analysis both experimentally and compared the results with Finite elemental outcomes. They have opted for SOLID 65 & SHELL 181 for nonlinear static analysis to bind concrete and Fibre-reinforced polymer. Four-point loading is carried out on the beam to derive Loading and deflection at the beam’s mid-span. The collation of results for the experimental and FEA methods shows only minuscule differences among the outputs.

Pradeep Singh et al.^14^ simulated RC beams in ANSYS Software, and the author solved concrete beam problems effectively. The effects of different fibre polymers reinforced the RC beam externally. They mainly carried to account for the strength necessary to withstand flexural and shear. ANSYS modelling was done for a simple RC beam, and fibre-reinforced polymer material laminate was used over the simple RC beam. Paraments like deflection on various loading conditions were concluded, understanding directional deflection and deriving Equivalent elastic strain. Saba Sabih et al.^15^ analysed concrete column structures, where the dynamic analysis predicted that the vertical column’s base area presumes more deflection. Some possible places or locations of deformations can be predicted using FEA Analysis through Ansys software. Jesse Santos et al.^16^ have used MEMS accelerometers in very different applications to monitor the dynamic forces and understand the fundamentals of earthquake force excitation signals. Hanchegmao^17^ et al. l. Made a detailed theoretical study on free and forced vibration where the propagation behaviour of a curved beam is analysed analytically, a comparison of vibration at different excitation levels is observed, and vibration excitation location or band is concluded.

New developments in material science have offered significant information on ways to enhance the usage and properties of concrete and cement composites. In concrete production, the early hydration reaction of calcium alumino-silicate hydrate (C-A-S-H) was investigated using the molecular dynamics simulations in Zheng et al.^19^. They found that elevated Al concentrations promote the rates of the hydration reactions, silicate chain polymerisation and the Qn cluster, (*n* ≥ 2) aggregation to provide atomic level information on how the Al/Si ratio influences the behaviour of the C-A-S-H gel^18^. Further, Zheng et al.^19^ also assessed the feasibility of incorporating an anionic WPU emulsion to enhance durability, resistance to hostile environments, and bonding strength in cement repair mortars. They confirmed that WPU emulsion formed a three-dimensional polymer network with hydration products^19,20^. The results of this study offer strategies for engineering polymer-modified repair materials in practice.

Simultaneously, researchers have developed new computational methods to study other structural responses and solicitations. In the engineering simulation domain, Wang et al. (2020) developed a meshless generalised Finite Difference Method (FDM) for stress analysis on three-dimensional composite elastic materials^21^. Similarly, Kabir et al.^22^ proposed a multi-step Bézier formulated for nonlinear vibrations and post-buckling analysis of GRFC beams, demonstrating a precisely stable approach to predicting the elastic constant^22^. These computational improvements can serve as an alternative to finite element analysis (FEA), utilizing experimental data to enhance understanding of the static and dynamic behaviours of reinforced concrete (RC) beams.

## Experimental and fem methods

### Experimental details

**Fig. 1:**
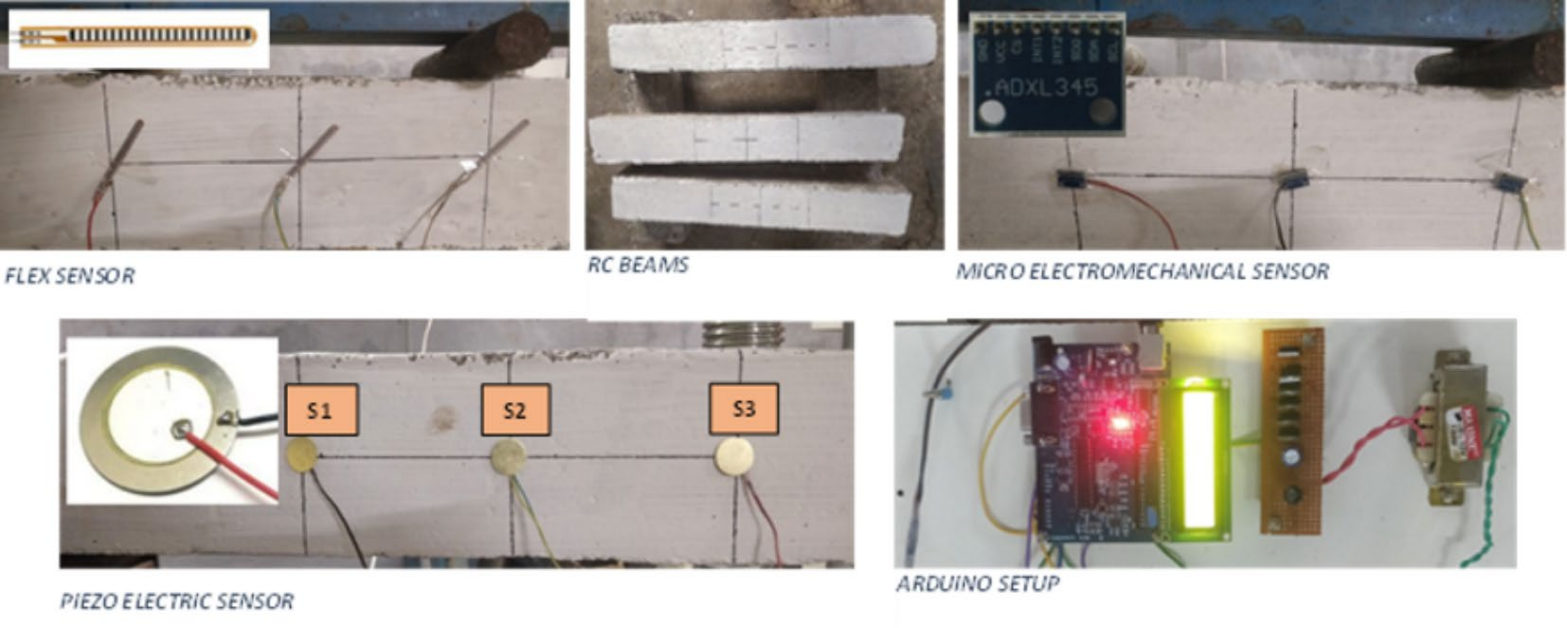
All the components and devices needed for the experimental setup.

First, the degrees of deflection are to be measured at three different locations, S1, S2, and S3, across the RC beam, respectively, as shown in (Fig. 1). Here, we have used a 5.588 cm long variable resistance flex sensor. The resistance caused by the ink placed inside the sensor will give proportionate results for deflection. Using this, we will find the deflection for varying load cases.

Likewise, the previous sensor, another sensor known as a microelectromechanical sensor, is a miniaturised device for observing the vibration occurrence at 3 locations. The RC beams for different load cases measure the amount of changes in the dimension of the geometry of the beam. The 3rd sensor used in our experiment is the Piezoelectric sensor placed over 3 locations similar to the other two sensors where PZS is subjected to deformation under load conditions, which is then interpreted in the voltage magnitude.

Along with these three sensors, one more sensor known as a force sensor (FRS) is deployed, which calculates the force exerted over the beam across various intensities of load steps. A force sensor is a typical transducer with an inscribed metal wire over the metal plate and is covered entirely inside polyimide film to avoid external disturbance. The load applied is magnified as an electric signal, and its proportionate reading is calibrated. The study prioritises the determination of sensors that fulfil specific requirements set for sequential static and dynamic force measurements in RC beams. The FRS was chosen due to its capacity for reliable force measurements at elevated load levels, while the FLS was selected because of its superior capabilities in the deflection measurement. The PZS was used because it can convert mechanical stress into electrical signals and is applicable in dynamic conditions. Finally, the MEMS accelerometer was picked because it provides higher-resolution vibration data, boosting the characerisation of the dynamic response.

**Fig. 2:**
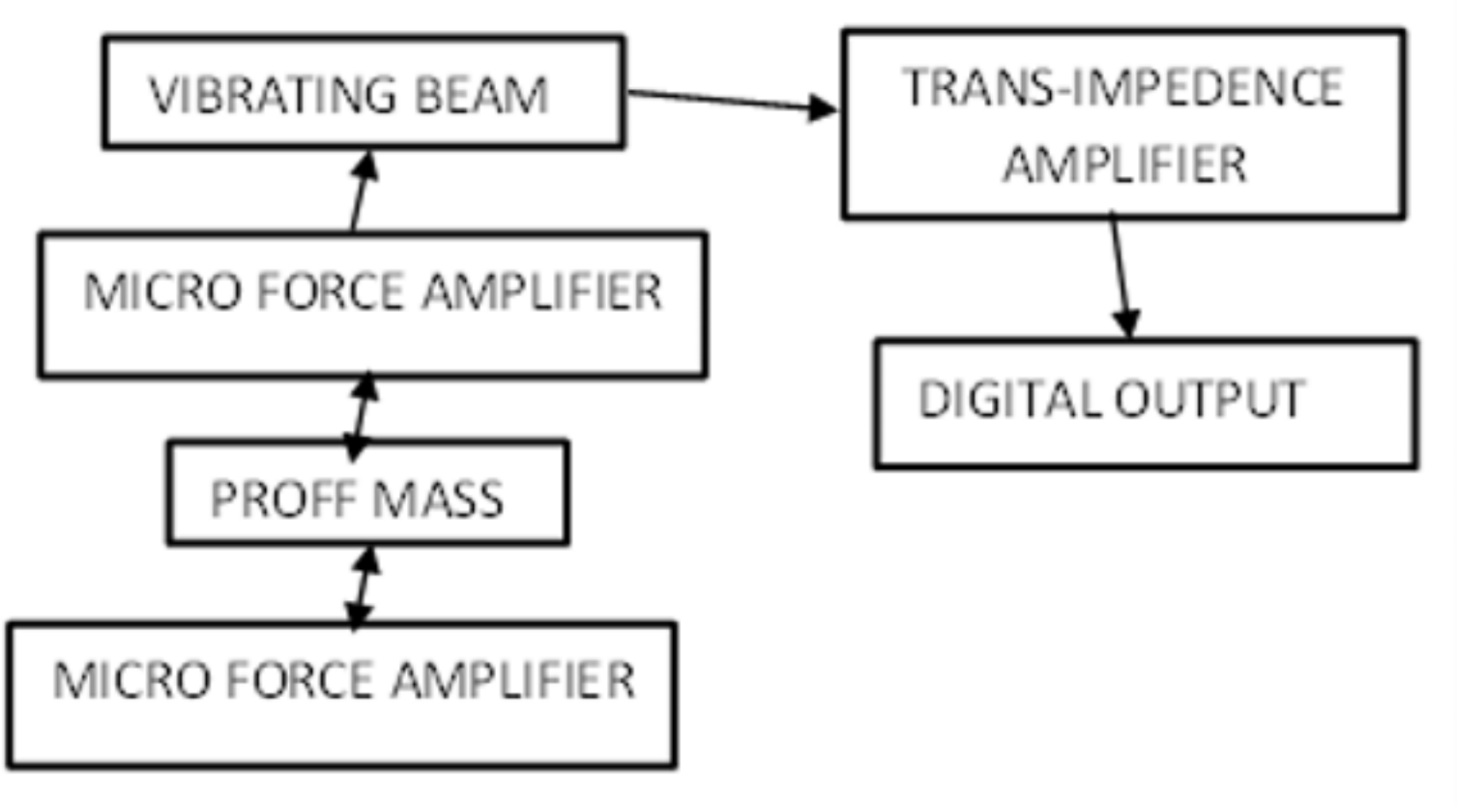
Block diagram representing the entire process flow with the sensor in reading the deformations in mm.

All four sensors are connected to the Arduino board, which shows the data findings through an open-source Arduino integrated development environment (IDE). A basic program is made to differentiate data from the sensors into dimensions and measures. With the help of the computer system, we can track live data in the form of measurements. The specifications of the RC beam are 1 m in length and 150 mm in breadth and depth. Marking is made across the beam at L/3(S1), L/2(S2) & 2 L/3 (S3); over these positions, the sensors are placed. In total, we have 4 RC beams that are similar. A block diagram illustrating the entire process flow can be seen in Fig. 2, which also includes a sensor that reads the deformations in millimetres.

The beam put under tensile force tends to undergo deformation, as noted by the sensor. The force is minimal in some cases, and a force amplifier is placed to amplify the intensity of deformation under various load conditions. Then, the proportionate reading can be seen from a personal computer using open-source software.

**Fig. 3:**
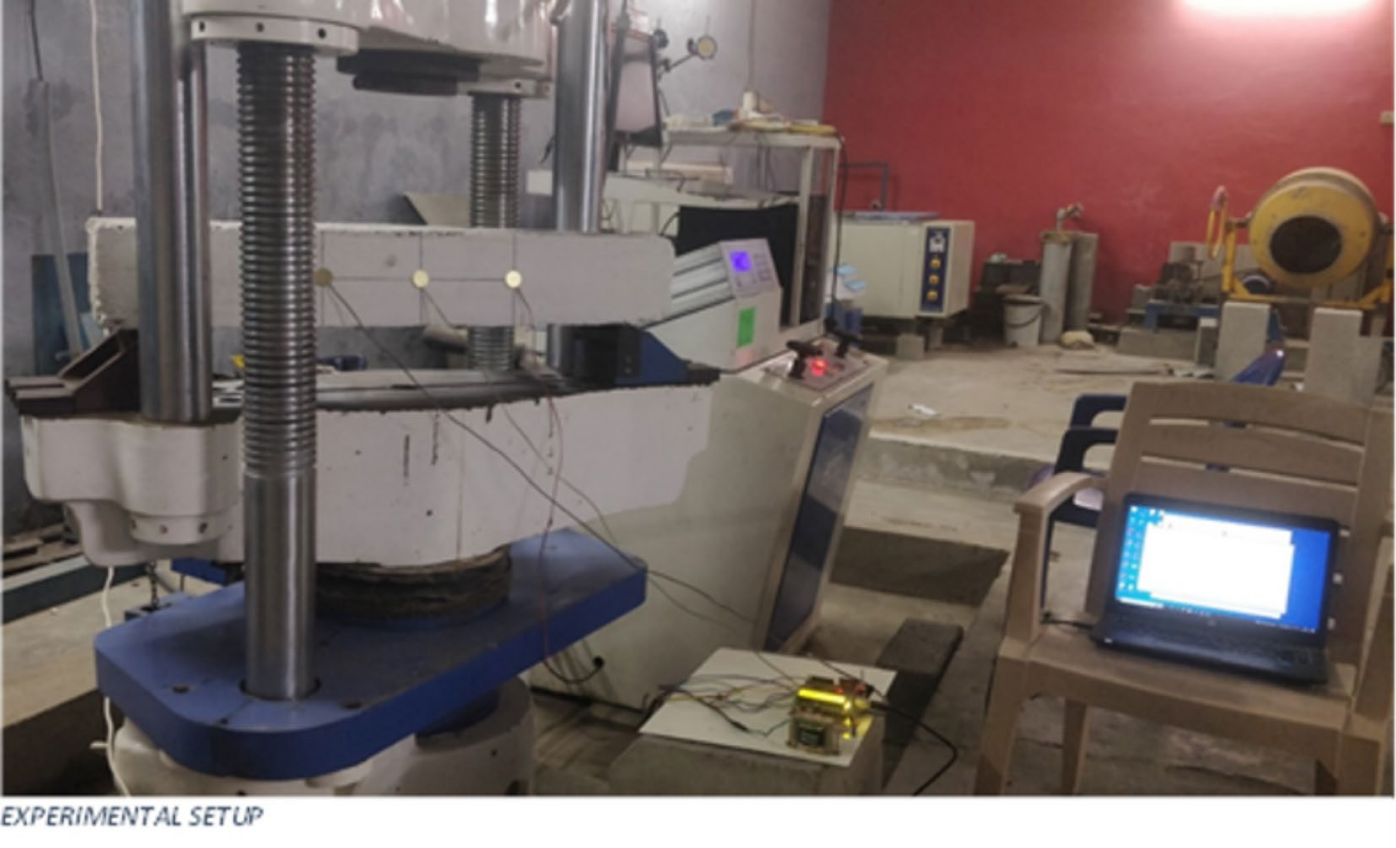
Experimental setup, with RC beam and Piezo sensor placed in S1–S3. Connected with the ARDUINO setup, the output can be noted from a personal computer.

All four types of sensors are placed at the marking at three positions externally, and the readings are observed and tabulated one by one depending on the load cases. The above image shows the experimental setup of the RC beam on the UTM, where a gradual load is applied from 10 to 100KN, starting from 1 to 10 min. Sensors placed at S1, S2& S3 read the geometric changes undergone during the experiment phase. The results are compared in the form of a graph with the FEM outcomes. The experimental setup shown in Fig. 3 places RC beams and piezo sensors in S1, S2, and S3. The output can be noted from a computer connected to the ARDUINO setup.

### FEM details

**Fig. 4:**
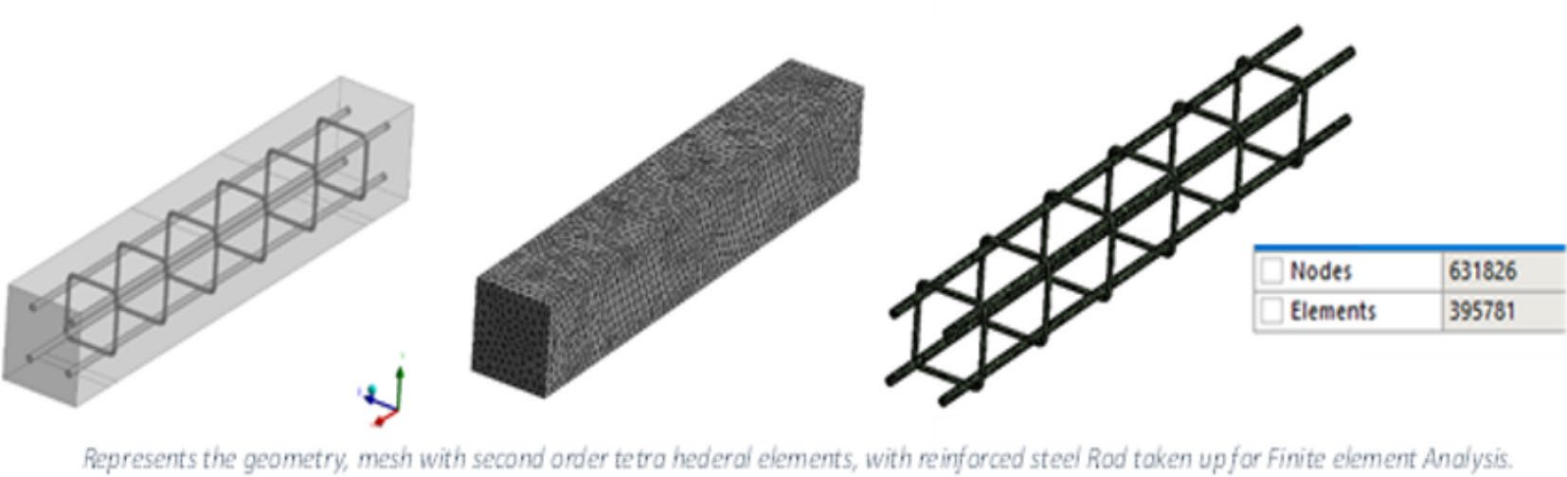
Ansys representation of RC beam, Mesh on RC beam, incorporated steel reinforcement.

**Fig. 5:**
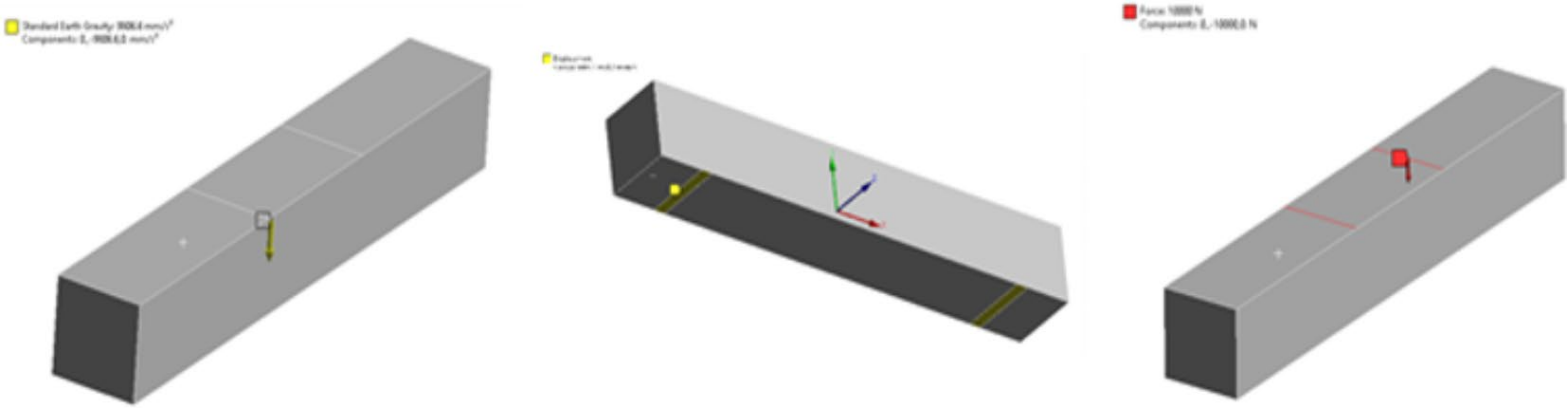
Represents the boundary conditions applied in Ansys FEM analysis along X, Y and Z axis.

The RC beams used for conducting the experiments were modelled and analysed in ANSYS 16.0 software accessed through the university’s academic licensing program for teaching and research purposes (www.ansys.com). The solid block was meshed using second-order tetrahedral elements to ensure accurate convergence of the results. Mid-surface casting and extruding were not considered because they are less efficient than batch meshing. Each aspect could move about three dimensions along the X, Y, and Z axis at each node. Half the beam was meshed and copied through the ANSYS tool to achieve equal spacing and distances. Material properties were given to the concrete and the reinforced bar, as indicated below in (Table 1). The model assumed standard material properties, including a Poisson’s ratio of 0.18 for M30 concrete and 0.3 for structural steel. These assumptions are consistent with typical material behavior under static loading. After that, the boundary conditions were set, and constraints to displacement were imposed. The mesh of the RC beam, the incorporated steel reinforcement, and the boundary conditions along the X, Y and Z axes are shown in the ANSYS representation in Figs. 4 and 5. In this FEM study, the load application was also subdivided into load steps. Time steps were also defined according to the load case conditions; the loads varied from 0 to 100 kN with increments of 10 kN, and the time steps varied from 0 to 10 min.

**Table 1:** Material properties of RC Beam components.

S.No	Name of the element	Material	Young’s modulus (MPa)	Density (Kg/m^3^)	Yield tensile strength (Mpa)	Poisson’s ratio
1	Reinforcement bars	Structural steel	210000	7850	500	0.3
2	concrete	M30	35000	2780	30	0.18

## Experimental outcomes

**Fig. 6:**
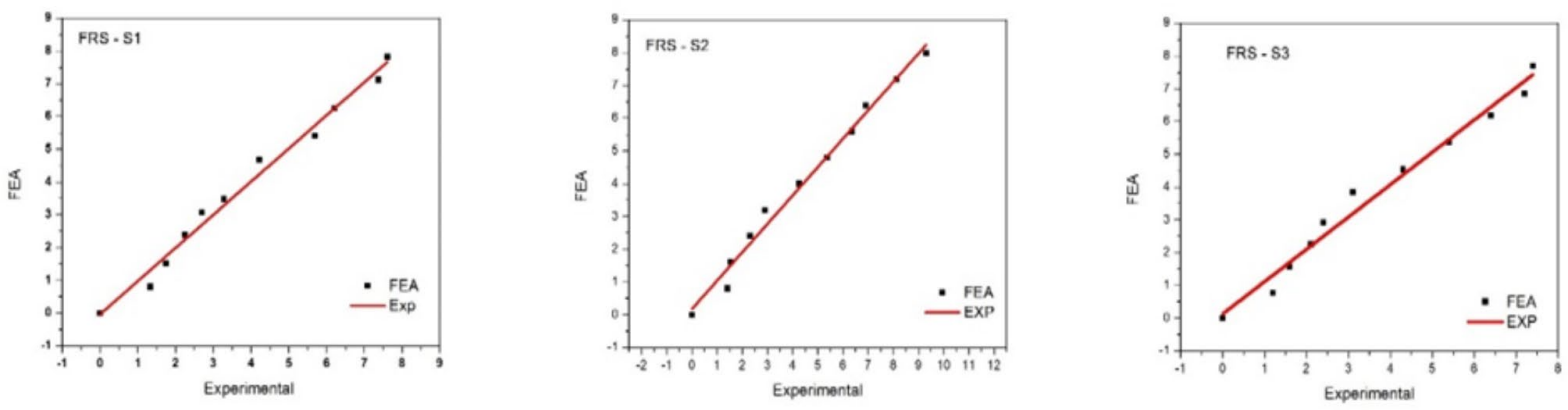
Graphical findings from FRS sensors measured at positions S1–S3.

The output of Fig. 6 from the force resistance sensor (FRS) indicates that the deviations in the experimental results correlate with the Finite Element Analysis (FEA) outcomes. It is observed that there are variations at different lengths across various step load cases. At the S1 location, the most significant discrepancy between the experimental and FEA results occurs at the initial load condition of 10 kN and the half-loading condition of 60 kN. At the S2 location, more deviation is noted in the early and final load cases of 10 and 100 kN, respectively. The S3 location (2 L/3) exhibits the least deviation between 10 kN and 100 kN load conditions. Table 2 compares the FRS deviations for static loading conditions between the experimental and analytical (FEA) results.

**Table 2:** FRS deviation comparison between experimental and analytical (FEA) results under static loading conditions.

Time	Load	FRS deviation comparison for experiment vs. analytical (FEA) under static loading condition									
		mm									
minutes	kN	Experiment (S1_E_)	Analytical (FEA) (S1_A_)	Total deviation S1 (S1_E_–S1_A_)	Experiment (S2E)	Analytical (FEA) (S2_A_)	Total Deviation S2 (S2_E_–S2_A_)	Experiment (S3_E_)	Analytical (FEA) (S3_A_)	Total deviation S3 (S3_E_–S3_A_)	Total deviation average (S1, S2, S3)
0	0	0.00	0	0	0.00	0	0	0.00	0	0	0
1	10	1.33	0.812	0.518	1.41	0.812	0.608	1.20	0.773	0.407	0.511
2	20	1.74	1.523	0.217	1.53	1.608	0.078	1.60	1.568	0.032	0.109
3	30	2.25	2.384	0.134	2.31	2.405	0.095	2.10	2.248	0.148	0.125667
4	40	2.70	3.081	0.381	2.90	3.201	0.301	2.40	2.926	0.526	0.402667
5	50	3.28	3.484	0.204	4.26	3.998	0.262	3.10	3.846	0.746	0.404
6	60	4.22	4.679	0.459	5.38	4.795	0.585	4.30	4.540	0.240	0.428
7	70	5.69	5.416	0.274	6.36	5.592	0.768	5.40	5.384	0.016	0.352667
8	80	6.21	6.254	0.044	6.91	6.388	0.522	6.40	6.189	0.211	0.259
9	90	7.37	7.120	0.250	8.14	7.185	0.955	7.20	6.842	0.358	0.521
10	100	7.61	7.818	0.208	9.31	7.981	1.329	7.40	7.695	0.295	0.610667
									**Total average %**		**0.338515**

**Fig. 7:**
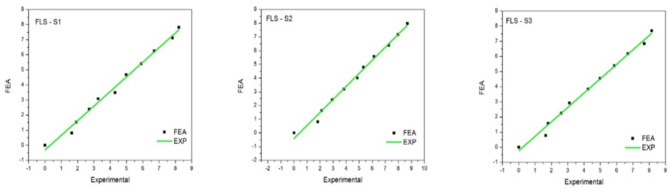
Graphical findings from FLS sensors measured at positions S1–S3.

Continuing with the flex sensor depicted in Fig. 7, the graphical representation at various locations indicates distinct variations. In the S1 region comparison, deviations are observed at the initial load of 10 kN, at half loading (50 kN), and at the final loading step of 100 kN. Observations at S2 reveal deviations at the middle and second-to-last load cases, precisely at 50 and 90 kN. In S3, deviations are only noted in the final load case.

**Table 3:** FLS deviation comparison between experimental and analytical (FEA) results under static loading conditions.

Time	Load	FLS deviation comparison for experiment vs. analytical (FEA) under static loading condition									
		mm									
minutes	kN	Experiment (S1_E_)	Analytical (FEA) (S1_A_)	Total deviation S1 (S1_E_–S1_A_)	Experiment (S2_E_)	Analytical (FEA) (S2_A_)	Total deviation S2 (S2_E_–S2_A_)	Experiment (S3_E_)	Analytical (FEA) (S3_A_)	Total deviation S3 (S3_E_ - S3_A_)	Total deviation average (S1, S2, S3)
0	0	0.00	0	0	0.00	0	0	0.00	0	0	0
1	10	1.64	0.812	0.828	1.83	0.812	1.028	1.65	0.773	0.857	0.904333
2	20	1.92	1.523	0.397	2.12	1.608	0.512	1.80	1.568	0.232	0.380333
3	30	2.71	2.384	0.326	2.93	2.405	0.525	2.60	2.248	0.352	0.401
4	40	3.26	3.081	0.179	3.84	3.201	0.639	3.10	2.926	0.174	0.330667
5	50	4.30	3.484	0.816	4.86	3.998	0.862	4.25	3.846	0.404	0.694
6	60	4.98	4.679	0.301	5.32	4.795	0.525	4.97	4.540	0.430	0.418667
7	70	5.91	5.416	0.494	6.14	5.592	0.548	5.86	5.384	0.476	0.506
8	80	6.70	6.254	0.446	7.27	6.388	0.882	6.68	6.189	0.491	0.606333
9	90	7.82	7.120	0.700	7.97	7.185	0.785	7.70	6.842	0.858	0.781
10	100	8.21	7.818	0.392	8.70	7.981	0.719	8.15	7.695	0.455	0.522
									**Total average %**		**0.50403**

Regarding FLS, the graph indicates more instances of deviation as we approach the final load case. Aside from a few exceptions, the comparison of results shows relatively little deviation overall. A more detailed output for various load cases can be found in (Table 3).

**Fig. 8:**
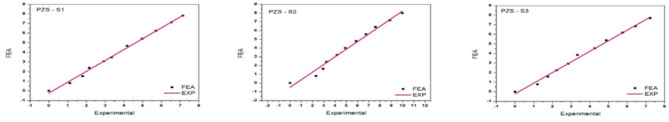
Graphical findings from PZS sensors measured at positions S1–S3.

When measurements were taken using the piezoelectric sensor (PZS) shown in Fig. 8, little deviation was observed at locations S2 and S3. However, in the S1 region, a significant deviation was noted at the 100 kN load case. These results are also reflected in (Table 4).

**Table 4:** PZS deviation comparison between experimental and analytical (FEA) results under static loading conditions.

Time	Load	PZS Deviation comparison for experiment vs. analytical (FEA) under static loading condition									
		mm									
minutes	kN	Experiment (S1_E_)	Analytical (FEA) (S1_A_)	Total deviation S1 (S1_E_–S1_A_)	Experiment (S2_E_)	Analytical (FEA) (S2_A_)	Total deviation S2 (S2_E_–S2_A_)	Experiment (S3_E_)	Analytical (FEA) (S3_A_)	Total deviation S3 (S3_E_–S3_A_)	Total Deviation Average (S1, S2, S3)
0	0	0.00	0	0	0.00	0	0	0.00	0	0	0
1	10	1.12	0.812	0.308	2.31	0.812	1.508	1.20	0.773	0.407	0.741
2	20	1.80	1.523	0.277	2.94	1.608	1.332	1.76	1.568	0.192	0.600333
3	30	2.15	2.384	0.234	3.26	2.405	0.855	2.23	2.248	0.018	0.369
4	40	2.93	3.081	0.151	4.18	3.201	0.979	2.85	2.926	0.076	0.402
5	50	3.35	3.484	0.134	4.92	3.998	0.922	3.34	3.846	0.506	0.520667
6	60	4.17	4.679	0.509	5.87	4.795	1.075	4.27	4.540	0.270	0.618
7	70	4.98	5.416	0.436	6.77	5.592	1.178	4.89	5.384	0.494	0.702667
8	80	5.72	6.254	0.534	7.60	6.388	1.212	5.75	6.189	0.439	0.728333
9	90	6.53	7.120	0.590	8.87	7.185	1.685	6.45	6.842	0.392	0.889
10	100	7.15	7.818	0.668	9.98	7.981	1.999	7.23	7.695	0.465	1.044
									**Total average %**		**0.601364**

When comparing the deviation of the FRS sensor to the other three sensors, the slightest deviation is shown. This deviation is minimal for both experimental analysis and FEM outcomes, with negligible differences in the results. The final sensor chosen for geometric monitoring of the reinforced concrete (RC) beam is a microelectrochemical system device, which measures the vibrations caused by various loading conditions.

**Fig. 9:**
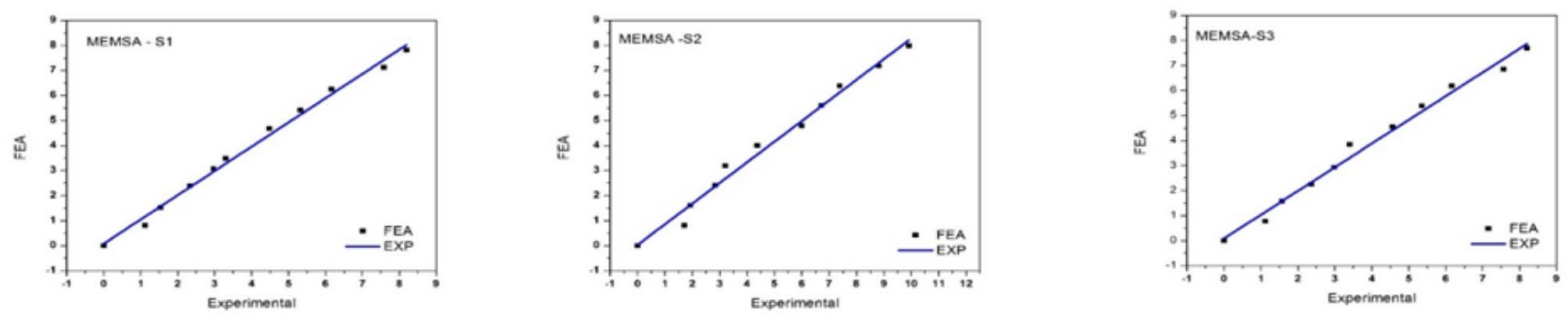
Graphical findings from MEMSA sensors measured at positions S1–S3.

An analysis of the deviation at location S1 indicates that there is very little difference between the experimental and FEM outcomes. In location S2, however, a higher deviation is observed at the 60 and 70 kN load cases. A similar pattern of deviation is apparent at location S3, where higher deviations are noted during the second-to-last and final load cases. Other causes of the deviations include environmental factors, calibration accuracy, and inherent sensor characteristics. For instance, the deviations of the PZS sensor were higher at S1 because of mechanical noises and ecological interference. On the other hand, the FRS sensor did not show much variation at all locations due to its calibration. The observed discrepancies between the sensor data and the FEA results can be mainly attributed to experimental and computational sources. Although all external factors like temperature variation and mechanical noise were controlled within the laboratory, the slight variations in the position and orientation of the sensors and calibration brought minor errors. For instance, the PZS sensor, because of its high sensitivity, performed slightly worse under higher load, which could be attributed to either signal saturation or slightly misaligned elements. Also, assumptions of FEA modelling include idealised material properties, and the model’s boundary conditions do not always reflect real-life experimental conditions, hence the differences observed. The graphical results from measurements taken with MEMSA sensors at positions S1, S2, and S3 are shown in (Fig. 9).

**Table 5:** MEMSA deviation comparison between experimental and analytical (FEA) results under static loading conditions.

Time	Load	MEMSA deviation comparison for experiment vs. analytical (FEA) under static loading condition									
		mm									
minutes	kN	Experiment (S1_E_)	Analytical (FEA) (S1_A_)	Total deviation S1 (S1_E_–S1_A_)	Experiment (S2_E_)	Analytical (FEA) (S2_A_)	Total deviation S2 (S2_E_–S2_A_)	Experiment (S3_E_)	Analytical (FEA) (S3_A_)	Total deviation S3 (S3_E_–S3_A_)	Total Deviation Average (S1, S2, S3)
0	0	0.00	0	0	0.00	0	0	0.00	0	0	0
1	10	1.12	0.812	0.308	1.71	0.812	0.908	1.12	0.773	0.327	0.514333
2	20	1.54	1.523	0.017	1.93	1.608	0.322	1.57	1.568	0.002	0.113667
3	30	2.33	2.384	0.054	2.84	2.405	0.435	2.36	2.248	0.112	0.200333
4	40	2.97	3.081	0.111	3.20	3.201	0.001	2.98	2.926	0.054	0.055333
5	50	3.30	3.484	0.184	4.37	3.998	0.372	3.40	3.846	0.446	0.334
6	60	4.48	4.679	0.199	5.99	4.795	1.195	4.56	4.540	0.020	0.471333
7	70	5.32	5.416	0.096	6.71	5.592	1.118	5.36	5.384	0.024	0.412667
8	80	6.16	6.254	0.094	7.38	6.388	0.992	6.16	6.189	0.029	0.371667
9	90	7.58	7.120	0.460	8.81	7.185	1.625	7.57	6.842	0.728	0.937667
10	100	8.19	7.818	0.372	9.91	7.981	1.929	8.20	7.695	0.505	0.935333
									**Total average %**		**0.395121**

Table 5 indicates that the MEMSA sensor experiences more significant deviation under high load conditions than low load conditions. Among the four sensors used in the experimental procedure, MEMSA and FRS exhibited minimal variation, demonstrating their precision in contrast to the PZS and FLS sensors. Under these conditions, The MEMSA and FRS sensors showed slight variation, and the mean error was 0.39% and 0.33%, respectively, which indicates their accuracy. On the other hand, the PZS sensor has shown higher deviations at high load conditions, which may be attributed to its higher sensitivity to geometric changes and alignment accuracy during the test. This goes a long way in underlining the need to be careful with calibration and positioning, even in laboratory experiments, to get the correct data. The total deviations observed in Tables 3 and 4 PZS, as well as in the FLS data, are 0.601 and 0.504, respectively. These values indicate that both cases exhibit more significant deviation than the FEA outcomes. This trend is also reflected in both the graphs and the tables.

**Fig. 10:**
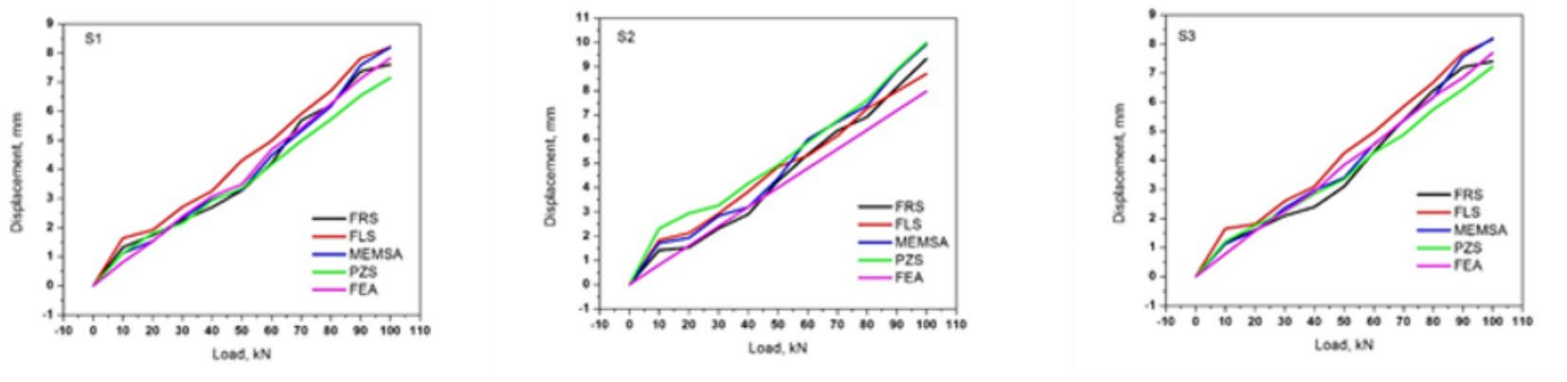
Total graphical results from various sensors measured at the S1–S3 positions.

Discuss each position and the outcomes presented in Fig. 10 using applied sensors and the FEA method. We begin with the S1 location, which is situated at a distance of L/3 from the edge of the RC beams. The graph shows the displacement of the load, with curves representing each sensor and their corresponding FEA outcomes.

In this context, the PZS curve exhibits significant deviation in interpreting the vibrations or deflections in the RC beam. Following this, we can see the FEA simulation outcomes for each time step. According to the FEA results, the MEMSA sensor shows the least deviation compared to the observed values, while the FRS sensor ranks second lowest in deviation. The best results from the S1 location are obtained using the FLS sensor. Overall, the data indicates that FEA, MEMSA, and FRS results are closely aligned, with very minimal deviation compared to the other sensors, FLS and PZS.

For the S2 location, located at L/2 along the beam, the PZS sensor had the most significant deviation among all sensors, and the MEMSA sensor had a more substantial deviation than the S1 location. The FRS sensor was the most accurate regarding the FEA outcomes, proving its efficiency. At S3, the readings were closer to those at S1, especially at 2 L/3 along the beam, and the least variation was recorded on FLS. During the incremental static loading in this study, which ranged from 10 kN to 100 kN, MEMSA sensors demonstrated high responsiveness and accuracy. These sensors effectively captured changes in deflection at points S1, S2, and S3, ensuring that the inputs for dynamic analysis were reliable. Thus, the MEMSA and FRS sensors were the most stable, and deviations in the results were barely noticeable at S1 and S3. Here, PZS and FLS had higher deviations and were unsuitable for this experiment. These results indicate that for economical static analysis, the FEM method in ANSYS is a viable substitute for experimental techniques, especially when using the enhanced accuracy of MEMSA and FRS sensors. The different mechanical responses incurred at these points can explain the variation in the sensor results obtained at positions S1, S2 and S3 along the beam. S1 and S3, those closer to the supports, showed deviations due to lower bending moments and boundary conditions. On the other hand, S2, at the mid-span, showed a maximum deflection and stress concentration. Furthermore, it is possible that variations in the properties of the local material and slight differences in the position of the sensors could also contribute to observed differences.

## Modal analysis to find natural frequency

Along with the static part of the study, it is necessary to know the dynamic behaviour of the RC beams to maintain and understand the failures due to cyclic loads for prolonged periods. Usually, the cycle over a structure is monitored by observing the Natural frequency. Knowing the structural failure and saving the structure at the right time is like learning it. It’s also prime to know about mechanical attributes responsible for materials’ behaviour to understand the dynamic characteristics. Young’s modulus, Poisson ratio and density are the essential attributes used in Ansys software.

**Table 6:** Modal shapes and corresponding natural frequencies (NF).

Mode shape	Natural frequency (Hz)
Mode shape 1	103.82
Mode shape 2	130.9
Mode shape 3	172.16
Mode shape 4	198.09
Mode shape 5	209.71
Mode shape 6	293.01

**Fig. 11:**
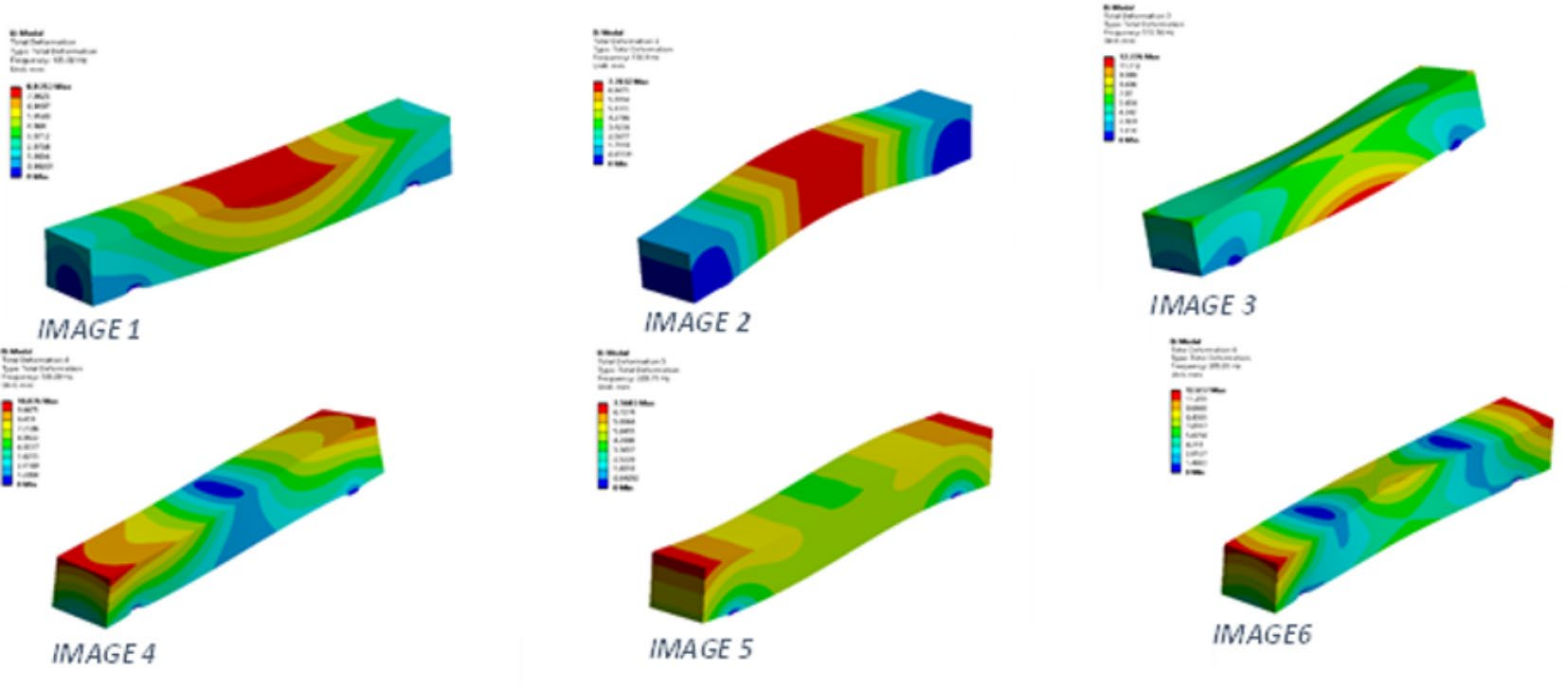
Simulation results for various mode shapes highlighting maximum deformation in mode shapes 3 and 6.

The outcomes observed after performing the modal analysis include the natural frequencies and mode shapes of the RC beam under load cases ranging from 10 kN to 100 kN. At 103.82 Hz (Table 6; Fig. 11 image 1), maximum vibration is observed at the centre above the neutral axis. At 130.9 Hz (mode 2), deformation is concentrated at the central region, making this mode particularly critical. At 172.16 Hz (mode 3, Fig. 11 image 3), the lower part of the central region exhibits maximum deformation under the applied frequency. For frequencies above 200 Hz, mode five at 209.71 Hz (Fig. 11 image 5) shows less significant deformation than mode 4. The extent of deformation is more localised in this mode compared to others.

Finally, at the maximum frequency of 293.01 Hz (mode 6), deformation is primarily concentrated in the upper corner regions along the length of the beam (Fig. 11 image 6). These corner areas are critical for addressing dynamic load cases, as deformation initiation could reduce the structure’s lifespan. Focusing on these regions can help mitigate dynamic effects and prolong the service life of RC structures.

**Fig. 12:**
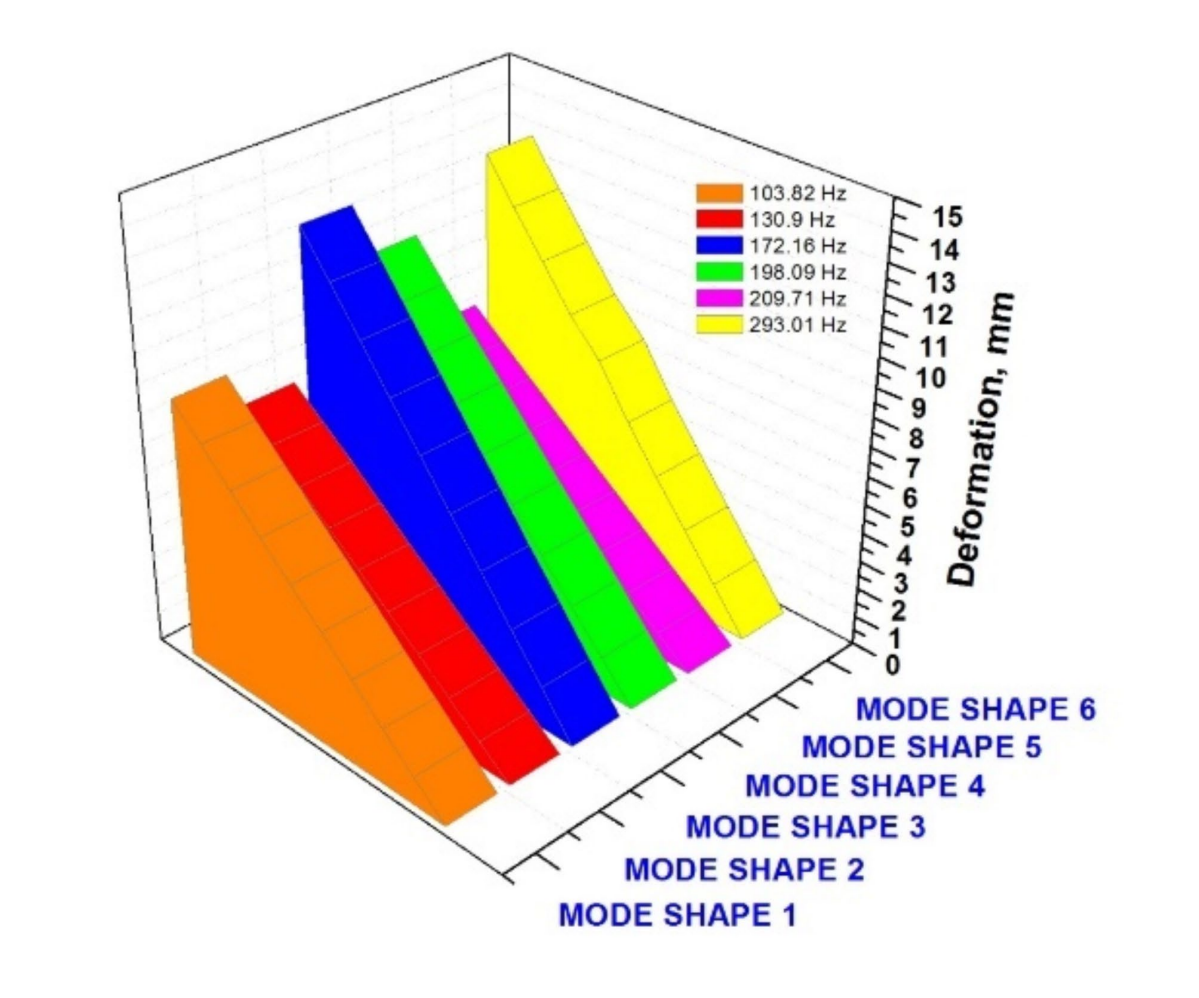
Mode shapes vs. deformation in mm.

From the above Fig. 12, comparing all the mode shapes in chart format yields that the deformation in mm is seen maximum in mode shape 6 with a frequency of 293.01 Hz followed by mode 3 of 172.16 Hz. These two specific mode shapes are seen to be high in deformations. Thus, it can be taken at this 2-frequency range in the RC structure (i.e.) at the upper corner ends and bottom centric portion. It has to be given special attention, and in these areas, there should be monitoring band improvisation is a must. The modal analysis identified natural frequencies of 172.16 and 293.01 Hz, corresponding to critical deformation regions. The harmonic response analysis utilised standard damping values to simulate realistic behavior under static loading.

## Harmonic response of the beam under the loadings (10–100KN)

After knowing the modal shapes, it’s also necessary to understand how the structure reacts due to repeated cyclic loads. The sustained harmonic behaviour of the RC beam is predicted by observing the harmonic response, and the steady-state response of the RC beam is analysed in a linear load-step manner. When simulation is carried out on structures’ response towards sinusoidal repeated loading, it gives the harmonic nature of the RC beam. The same is applied at different intervals of step load conditions from 10kn to 100kn. Interpretation of harmonic Responses is the most essential thing in monitoring RC beams.

The simulation results predict the sustained dynamic behaviour of the beam in each load condition. The deflection sustained in every load condition increases with respect to the higher load condition. At 100KN load condition, the strain sustained is maximum. The transformation of fringe over the beam gives a clear visual of the harmonic transformation in the form of deviation captured in mm.

Following the outcomes listed in the tabulation also gives the same significant results on deviation sustained at each location S1–S3. Table 7 shows that the same resembles the modal shape outcomes. Again, here, the corners of the beam undergo high fluence under loading. Hence, it can be listed as a critical area.

**Fig. 13:**
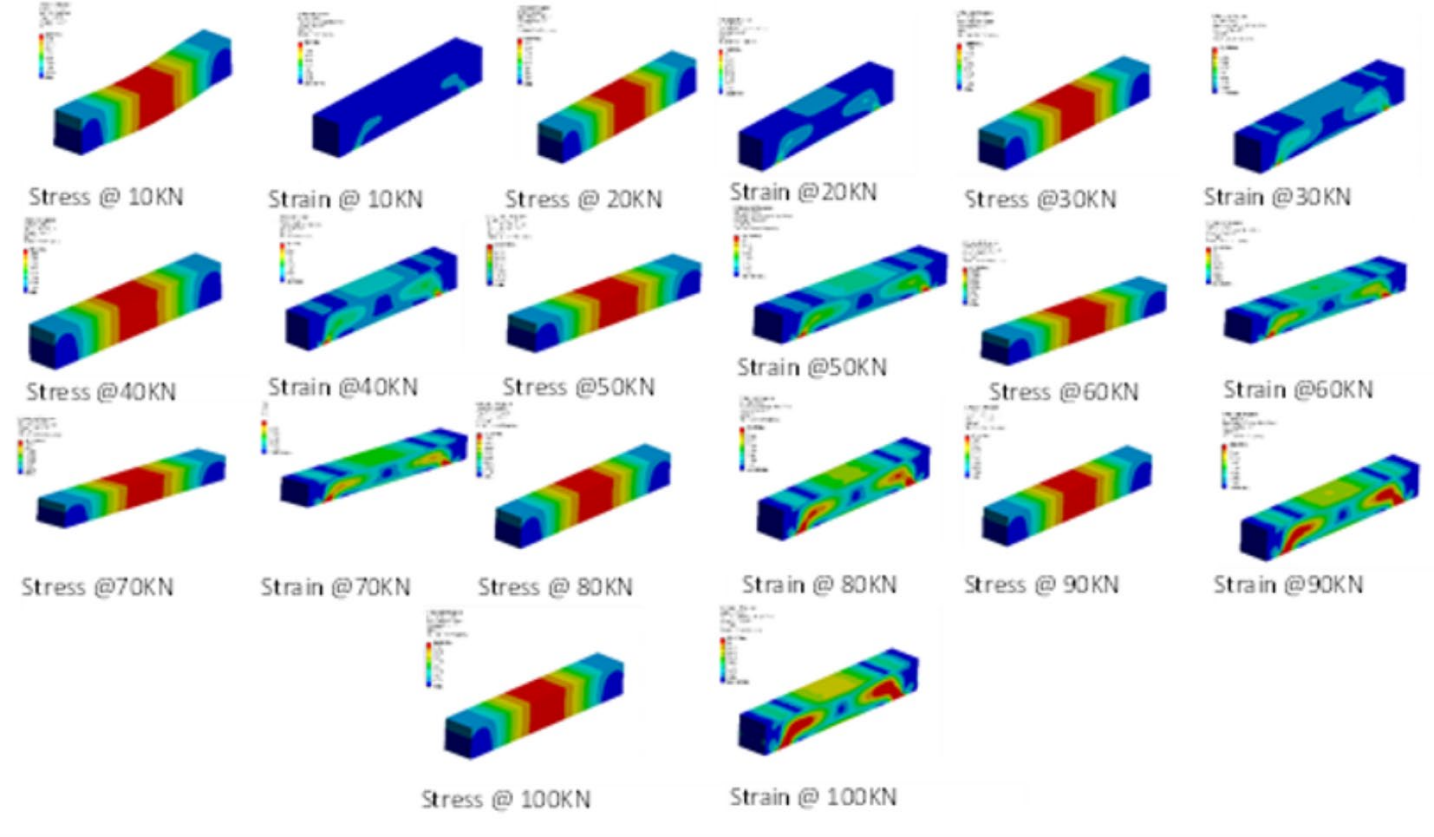
Harmonic response simulation captured at various load cases showing proportionate strain deformities.

In Ansys, we apply Young’s modulus, poison ratio, and mass density as inputs for material behaviour. These are the parameters primarily used to know harmonic responses. Apart from these parameter attributes, it is also a must to change some of the settings in Ansys software, starting with the state, which must be fully defined; the maximum range set should be 5000 Hz with 10kn step loading till 100kn. The solution method applied is mode supper portioning, and finally, the frequency range is program-controlled. The steady-state response of the RC beam subjected to sinusoidal concerning increasing load and time conditions can be seen in (Fig. 13).

1 $$[{\text{M}}\} \left\{ {{\text{u}}^{{..}} } \right\} + \left[ {\text{c}} \right]\left\{ {{\text{u}}^{.} } \right\} + \left[ {\text{k}} \right]\left\{ {\text{u}} \right\} = {\text{F}}_{0} {\text{sin }}({\text{wt}})$$

**Table 7:** Total deformation under harmonic loading.

LOAD (KN)	S1 (mm)	S2 (mm)	S3 (mm)
10	4.53	4.49	4.41
20	9.15	9.1	9.012
30	13.76	13.71	13.69
40	18.35	18.31	18.3
50	22.87	22.84	22.79
60	27.54	27.49	27.48
70	32.14	32.01	31.95
80	36.89	36.79	36.70
90	41.49	41.41	41.38
100	46.01	46.01	45.96

Either end S1 and S3 on the RC beams have been found to have higher deviation than the centre portion of the beam.

**Fig. 14:**
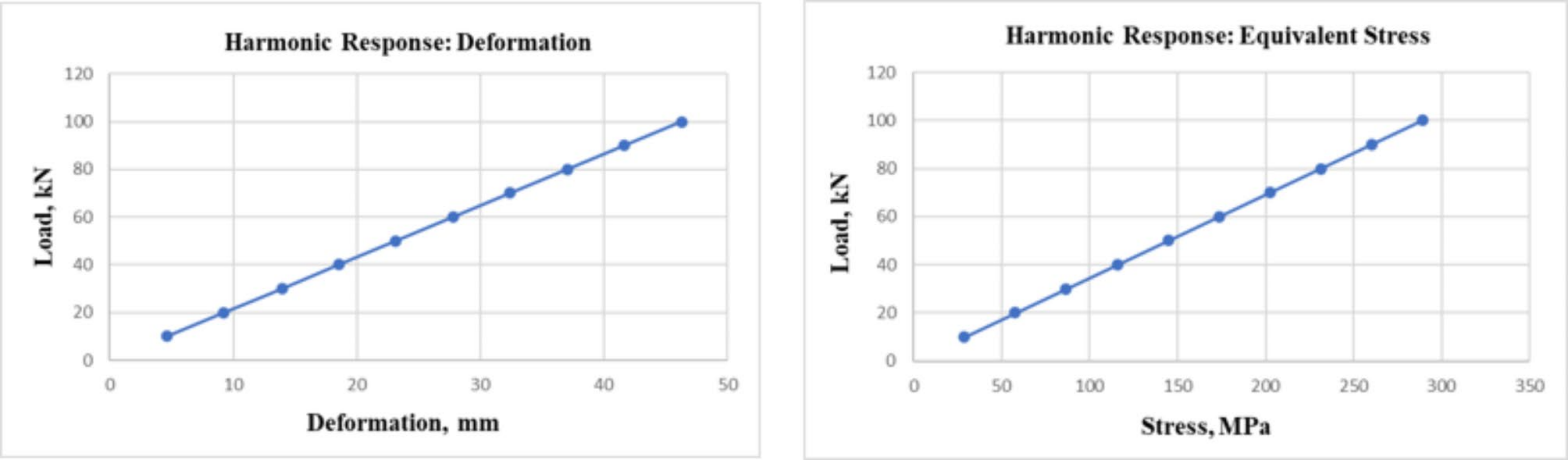
Graphical representation of load vs. deformation and load vs. stress plotted during dynamic loading.

Figure 14 shows the harmonic response observed in the form of load, gradually applied by monotonic loading over the RC beam, which devotes a steady and linear increase in the deformation, considering the RC beam to be isotopic in nature and linear-elastic until failure.

Initially, at the early load condition around 10KN, the deformation is seen in the form of a deflection measuring 4.63 mm. At the middle range loading at 50KN, the deformation is around 23.15 mm. While observing the last load step at 100KN, the total deformation is noted to be around 46.3 mm. The total deformation undergone is 46.3 mm, which states that the RC beam will rupture upon such a peak load condition.

The second graph from Fig. 14, load vs. stress, signifies the amount of internal resistance exerted by the beam for constructive load case scenarios. In this slop of the line segment, joining all the points suggests that for 10KN, the starting condition expressed is 28.91 MPa. in the middle loading case. The equivalent stress observed at half load case is 144.56 MPa; following these last steps, loading is 289.12 MPa. The harmonic study response suggests that as the load increases, it’s evident that the RC beam is at Risk. So, to avoid such a problem, it’s good to add countermeasures.

## Conclusion

In this study on RC beams, we have seen a comparison of various sensors and FEA results. We analysed the beam in both static and dynamic conditions. Finally, some future scopes for better observation will be discussed below.

i. The RC beam is tested experimentally in the UTM machine, and observations are made using the sensors placed in various locations; for the same beam, the FEA analysis is conducted using Ansys software. The best results, along with the FEA outcomes, are MEMS and FRS. In contrast, FLS and PZS have higher deviations than the other two sensors. In this case, it can be concluded that instead of going for higher expense testing and applying sensors, it’s good to take simulation analysis from the FEA method, saving time and capital.
ii. The comparison of FEA and experimental outputs from graph and tabulation yield provides sufficient validation to suggest that the FEA methodology provides results equivalent to the experimental values. Thus, instead of going for experimental analysis, time and capital can be saved by opting for the FEA methodology.
iii. In the second part, the mode shape observations on the various frequency ranges suggest that mode shape six and mode shape 3, with 293.01 Hz and 172.16 Hz, are the two frequency ranges where the deformation is observed to be much higher than that of other mode shapes.
iv. The harmonic response of the RC beam excited for different ranges of load conditions has been studied. Variation in deflection caused by the frequency amplitude best characterises the vibration response of a component subjected to dynamic loading. With the increase in load, the deflection also increases correspondingly. The corners of the beam are at risk, where deformation may be initiated.
v. The futuristic aspect of increasing the precision and accuracy of monitoring RC structures can be effectively done by using UV camera analysers. With such high-end tools, it is possible to tell the crack initiation time and other dimensioning information about the cracks. Even live updates on residual stress, stress relieving, binding strength, and creep can be directly monitored.
vi. The above observation shows how significant the monitoring methods are in telecasting the appropriate calibration of changes at various load cases. The extent of work can also be used to see the uneven surface and blow holes caused during curing itself.
vii. Future research could use machine learning techniques to process signals from the sensors and identify areas of weakness in RC structures. These models can also enhance the performance of structural health monitoring by recognising patterns and abnormalities in real-time.
viii. Research can be further developed to examine the application of innovative materials such as shape memory alloys or self-healing concrete in conjunction with intelligent sensors. This could also improve the durability and flexibility of RC structures under dynamic loading situations.
ix. This study may be used as a reference for analysing the static and dynamic behaviour of RC beams. The validated FEA methodology provides an efficient way of determining structural performance compared to expensive experimental methods practical in designing and retrofitting civil structures. These concepts can be used to assess other important RC structures like bridges, buildings, and other load-bearing members.

## Data Availability

The datasets generated and/or analysed during the current study are not publicly available due to privacy concerns but are available from the corresponding author on reasonable request.
